# Microbiome differences between trauma- and caries-derived periapical lesions using next-generation sequencing

**DOI:** 10.1080/20002297.2025.2560016

**Published:** 2025-09-23

**Authors:** Jiyuan Zhan, Yinxue Huang, Xinhui Meng, Yiquan Wang, Jia Liang, Fengjiao Zhu, Rui She, Shanshan Huang, Lijun Huo

**Affiliations:** Yunnan Key Laboratory of Stomatology & Department of Cariology, Operative Dentistry and Endodontics, The Affiliated Stomatology Hospital, Kunming Medical University, Kunming, China

**Keywords:** Trauma teeth, periapical lesions, microbiome, next-generation sequencing, campylobacter

## Abstract

**Background:**

While the microbiome of caries-derived periapical lesions has been extensively characterized, the microbial profile of trauma-derived periapical lesions remains poorly understood. This study aimed to characterize the apical microbiome of trauma-derived periapical lesions and identify taxonomic differences between trauma- and caries-derived periapical lesions.

**Methods:**

Twenty patients with periapical lesions were enrolled, comprising 10 trauma-derived cases (trauma group) and 10 caries-derived cases (caries group). Microbial samples were collected using sterile paper points inserted into the root canal exudate, followed by DNA extraction and Illumina sequencing of the hypervariable V3–V4 regions of the 16S rRNA gene. Bioinformatic analyses included *α*-diversity, *β*-diversity based on Bray–Curtis distance and differential abundance testing (LEfSe method with LDA score ≥ 2.0).

**Results:**

Sequencing revealed 36 bacterial phyla and 587 genera across all samples. Trauma group showed significantly greater relative abundance of *Campylobacter* (*P* = 0.002) compared to caries group, whereas *Prevotella* (*P* = 0.008), *Vibrio* (*P* = 0.041) and *Filifactor *(*P* = 0.006) exhibited reduced abundance. The core microbiota in the trauma group included *Phocaeicola*, *Porphyromonas* and *Pyramidobacter*, based on relative abundance. LEfSe analysis identified *Campylobacter* as a biomarker for the trauma group.

**Conclusions:**

Trauma-derived periapical lesions exhibited reduced microbial diversity compared to caries-derived periapical lesions, with *Campylobacter* identified as a potential pathognomonic taxon for trauma-derived periapical lesions.

## Introduction

Bacterial infection constitutes the fundamental etiological driver of apical periodontitis development [1]. Dental trauma induces vascular compromise, leading to pulp necrosis, thereby disrupting the pulp's innate immune responses against microbial invasion, including odontoblastic barrier function and phagocytic activity [2]. Notably, bacterial infiltration through patent dentinal tubules or enamel cracks can occur within 2−3 weeks post-trauma [3]. Maxillary anterior teeth demonstrate the highest trauma susceptibility, accounting for 78−96% of dental injuries reported in epidemiological studies [4]. Consistent with this pattern, a retrospective analysis of apical microsurgery cases found traumatic etiology in 58.4% of anterior teeth requiring endodontic intervention [5]. This raises a critical clinical question: do trauma-derived periapical lesions demonstrate equivalent microbiological profiles and pathogenic mechanisms to those arising from chronic carious exposure?

Contemporary metagenomic studies have established that endodontic infections constitute polymicrobial communities characterized by significant interindividual heterogeneity [6–8]. Emerging evidence suggests functional redundancy among distinct microbial consortia, where phylogenetically diverse communities may achieve comparable virulence potential through conserved metabolic pathways [9]. Nevertheless, the underlying pathogenic mechanisms can vary significantly across distinct microbial consortia. Microbial communities engage in complex ecological interactions mediated by quorum-sensing systems, metabolite exchange networks and antimicrobial metabolite production. These interspecies communications ultimately drive adaptive restructuring of community functional architecture [10]. Caries-derived periapical lesions demonstrate anaerobic predominance, with core microbiota comprising *Porphyromonas*, *Prevotella*, *Clostridium* and *Streptococcus* species [11]. Although the microbiome of trauma-derived periapical lesions remains underexplored, a seminal culture-based study of post-traumatic pulp necrosis revealed that obligate anaerobes predominating with *Micrococcus fine* (50%), *Propionibacterium acnes* (45%), *Clostridium nucleatum* (30%), *Prevotella buccalis* (30%) and *Fusobacterium longum* (25%) constituting core pathogens [12]. A similar study isolated and cultured microorganisms from teeth with intact crowns after trauma, including *oral Streptococci* (31%), *α-hemolytic Gram-positive cocci and α-hemolytic Gram-positive bacilli* (52%), *Staphylococcus spp.* (6.7%) and *sporulated Gram-positive Bacilli* (10.3%) [13].

Notably, accumulating evidence confirms that bacteria are not restricted to intraradicular spaces but can translocate through the apical foramen to colonize periapical tissues, forming extraradicular microbial communities. A study focusing on persistent apical periodontitis systematically characterized microbial communities in both intraradicular and extraradicular infections, identifying diverse bacterial species in periapical tissues, including *Porphyromonas*, *Prevotella* and *Fusobacterium* [14]. Similarly, next-generation sequencing analysis of paired root apices and periapical lesions revealed distinct bacterial communities in periapical lesions, with species such as *Dialister invisus* and *Filifactor alocis* showing significant association with clinical signs of persistent inflammation [15]. While most periapical infections originate from intraradicular biofilms, apical microenvironmental shifts, including hypoxia gradient formation, nutrient enrichment and immunocompetent cell infiltration, serve as ecological drivers for distinct microbial community structures between intraradicular and extraradicular infections [7].

Traditional culture-based methods have inherent limitations in cultivating >50% of oral microbiota due to fastidious growth requirements and viable-but-non-culturable (VBNC) states [11,16]. In response to these constraints, next-generation sequencing (NGS) platforms have become the gold standard for comprehensive microbial profiling. Compared to conventional culturing, NGS achieves 97.3% taxonomic resolution, enabling comprehensive detection of low-abundance taxa (<0.01% relative abundance), identification of uncultivable pathogens, characterization of new and potentially pathogenic species, while maintaining sensitivity for non-viable bacterial genomic remnants [17]. 16S rRNA gene sequencing, leveraging the conservation and variability of its sequence across species, enables efficient identification of microbial taxa in samples and comprehensive profiling of community composition. Among its hypervariable regions, the V3–V4 region, with its moderate length (approximately 460 bp), high coverage and compatibility with Illumina short-read platforms, has become a widely used amplification target in microbial community diversity analysis [18,19].

While the microbiota profiles of caries-derived periapical lesions have been well-characterized [20], the taxonomic signatures and virulence determinants of trauma-derived periapical lesions remain unclear. Comparative analysis of microbial communities between these two etiological types may reveal distinct environment-driven pathogenic mechanisms and identify potential therapeutic targets specific to trauma-derived periapical lesions. Therefore, this study aims to systematically compare the microbiome composition between trauma- and caries-derived periapical lesions using 16S rRNA gene sequencing, quantify taxonomic divergence and identify keystone taxa in trauma-derived periapical lesions, providing a foundation for developing precision endodontic therapies targeting lesion-specific keystone pathogens.

## Materials and methods

### Subject recruitment

The study was approved by the Medical Ethics Committee of the Stomatological Hospital of Kunming Medical University (KYKQ2022MEC002), and informed consent was obtained from the patients. Twenty patients diagnosed with chronic apical periodontitis were selected for this study. These patients presented to the Department of Endodontics at the Stomatology Hospital of Kunming Medical University from September to December 2023. However, patients with systemic diseases, those who had taken antibiotics or hormonal drugs in the previous three months, or pregnant patients were excluded from the study. All included teeth showed evidence of periapical hypodense shadows on imaging examinations, unresponsive pulp temperature tests and unresponsive pulp electrical vitality tests – specifically, patients had no perception or pain from thermal or electric stimuli. There was no history of endodontic treatment, sinus tracts or attachment loss. Radiographs also revealed no significant alveolar bone resorption. The screened affected teeth were categorized into two groups. The first group, named the trauma group (*n* = 10), included teeth suffering from chronic apical periodontitis due to trauma. Specifically, this group consisted of teeth with occlusal trauma, a history of trauma or orthodontic treatment and intact crowns without pulp-communicating defects or caries. The second group, the caries group (*n* = 10), consisted of teeth with chronic apical periodontitis caused by caries. Specifically, this group included teeth with significant pulp-communicating defects, such as deep caries or secondary caries. Moreover, these teeth had no history of occlusal trauma, traumatic injury or orthodontic treatment.

### Sample collection

After selecting teeth that met the criteria, endodontists obtained bacterial samples from the root canals following strict aseptic procedures. The specific operations are as follows:

The teeth were tightly isolated using rubber dam kits and dental floss to ensure that there was no communication between the surgical area and the oral environment. The rubber dam, dam clamps and tooth surfaces were disinfected with iodophor. All instruments, including high-speed handpieces, burs, files, root canal measuring rulers, paper points, forceps and irrigation needles, were autoclaved.

For teeth in the trauma group, after disinfection, access cavities were prepared directly using high-speed handpieces and burs. For teeth in the caries group, after removing carious tissues or old fillings, the teeth and rubber dam surfaces were disinfected again with iodophor before access cavity preparation.

The working length (WL) was accurately measured with the assistance of an apex locator. Hand files were used to negotiate and shape the root canals strictly according to the WL until size #25, preventing instruments from extending beyond the apical foramen. Sterile normal saline was used to irrigate the root canal orifice with a sterile needle, and the middle and upper segments of the root canal were dried with conventional sterile paper points.

Using forceps and a root canal measuring ruler, a sampling length of (WL + 3 mm) was marked on sterile absorbent paper points (size #20, 02 taper). Five such marked ultra-fine paper points were sequentially used to absorb exudate from the apical region and beyond the apical foramen, extending 3 mm beyond the WL. It was ensured that the paper points did not come into contact with any objects other than forceps and measuring rulers from the time they were removed until insertion into the root canal. During the operation, the paper points were advanced slowly; if significant resistance was encountered, the operation was stopped immediately to prevent damage to periapical tissues due to force. After removal, the paper points were checked to ensure that they were free from bending, creasing or deformation, then immediately placed into 2 ml cryovials and stored in liquid nitrogen. Additionally, if the paper points were observed to be contaminated with blood, the sample was discarded.

### Genomic DNA extraction and 16S rRNA gene sequencing

Genomic DNA was extracted from the samples using the CTAB method. The purity and concentration of the DNA were determined with a NanoDrop 2000 spectrophotometer, and the integrity of the DNA was evaluated via 1% agarose gel electrophoresis. Subsequently, the V3 and V4 variable regions were amplified by polymerase chain reaction (PCR) using the Phusion® High-Fidelity PCR Master Mix with GC Buffer (New England Biolabs). The upstream primer was 341F (5′-CCTAYGGGRBGCASCAG-3′), and the downstream primer was 806R (5′-GGACTACNNGGGGTATCTAAT-3′).

The PCR products were divided into individual aliquots based on their respective concentrations. The products were then purified by agarose gel electrophoresis using 2% agarose in 1 × TAE buffer. The target bands were subsequently extracted using the Universal DNA Purification and Recovery Kit.

The library was constructed using the NEB Next® Ultra DNA Library Prep Kit, and the resulting library was tested and quantified by qPCR with an Agilent 5400.

### Sequence analyses

All raw input sequences from all samples were processed using the DADA2 plugin (version 1.22.0) in QIIME2, which included quality control, denoising (to correct sequencing errors), merging and chimera removal to generate amplicon sequence variants (ASVs).

For quality control, 23 and 26 bases were truncated from the 5′ end of forward and reverse sequences, respectively. Reads were inspected from the 5′ to 3′ end: the first base with a quality score of ≤2 and all subsequent bases were truncated; sequences were discarded if their remaining length was shorter than the specified retention length (249 bp for both forward and reverse sequences).

Denoising was performed independently for each sample, with 1,000,000 reads used to train the error model.

For sequence merging, the forward primer (CCTAYGGGRBGCASCAG) and reverse primer (GGACTACHVGGGTWTCTAAT) were used, and successful merging required an overlapping region between forward and reverse sequences greater than the 12 bp threshold.

For chimera removal – performed following denoising with independent detection for each sample – DADA2 uses the default ‘consensus’ method: a chimera is flagged and removed only if detected in at least two samples.

The representative sequences of ASVs were queried against the pre-trained Greengenes2 database (trimmed to the V3V4 region flanked by the 338F/806R primer pair) using QIIME2's feature-classifier at a 99% similarity threshold to derive species annotations. Based on these annotations, ASVs and their constituent sequences annotated as chloroplasts, mitochondria, or unannotated at the kingdom level were excluded. Subsequently, the proportion of sequence counts per sample relative to the total was computed at the kingdom, phylum, class, order, family, genus and species levels to evaluate species annotation resolution across samples.

### Statistical analysis

For alpha diversity analysis, an intergroup species difference test was conducted to assess the normality and variance homogeneity of the data, using IBM SPSS V25.0. The results indicated that the data did not follow a normal distribution and that the variances were not homogeneous. Subsequently, a Mann-Whitney U test was applied to determine the significance of the observed differences. The obtained *p*-value was less than 0.05, suggesting that the difference was statistically significant. The similarity of microbial communities among samples was evaluated by principal coordinate analysis (PCoA) based on Bray–Curtis distance, weighted UniFrac distance and unweighted UniFrac distance, while the similarity of microbial communities between the two groups was evaluated using a PERMANOVA test.

## Results

### Study population

Table 1 depicts the demographic and clinical characteristics of the participants, including age, gender, tooth position and pain symptoms. Among these variables, the mean age was 27.4 years in the trauma group and 37.9 years in the caries group. A statistically significant difference in age was observed between the two groups (*P* = 0.032, *P* < 0.05). No statistically significant differences were observed in gender, tooth position or pain symptoms between the groups.

**Table 1.** Description of samples characteristics of trauma and caries group.

	Trauma group (*n* = 10)	Caries group (*n* = 10)	*P*-value
Age (mean)	27.4	37.9	0.032
Gender, % female	40.0	60.0	0.371
Tooth position, % front	80.0	60.0	0.355
Pain, % positive	90.0	50.0	0.057

Table 1 presents a comparison of distribution differences in age, gender, tooth position and pain symptoms between the trauma group (*n* = 10) and the caries group (*n* = 10), with specific statistical tests applied to analyze inter-group differences. For continuous variables (age), the Mann‒Whitney U test was used due to non-normal distribution; for categorical variables (gender, tooth position and pain symptoms), the chi-square test was employed. Statistical significance was defined as *P* < 0.05.

### ASV analysis of microbial communities

**Figure 1.**
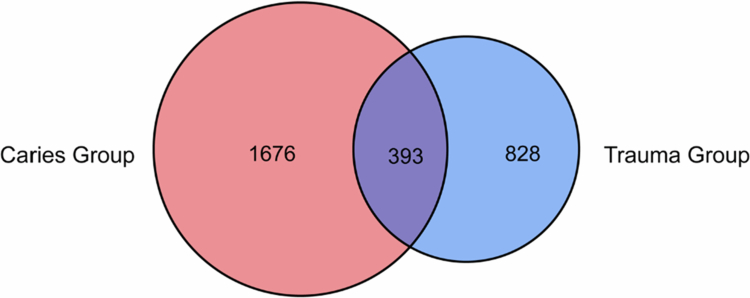
Venn plot of microbial communities in caries group and trauma group.

In this study, 10 bacterial samples from both the inner and outer regions of the apical areas of caries-derived periapical lesions and trauma-derived periapical lesions were collected for 16s rRNA sequencing analysis, and a total of 3,427,817 original sequences were obtained. After quality control, denoising, splicing and dechimerization of the raw sequences, 3,014,582 high-quality sequences were acquired. Specifically, 1,549,458 sequences were derived from the caries group, whereas the trauma group yielded 1,465,124 sequences. On average, each sample contained 150,729.1 sequences, with an average sequence length of 423.16 base pairs. Figure 1 illustrates that a total of 2069 ASVs were identified in the caries group, among which 1676 ASVs were exclusive to this group. In contrast, the trauma group had 1221 ASVs, with 828 of which were unique. Overall, the two groups exhibited a total of 393 shared ASVs.

### Microbial community composition

**Figure 2.**
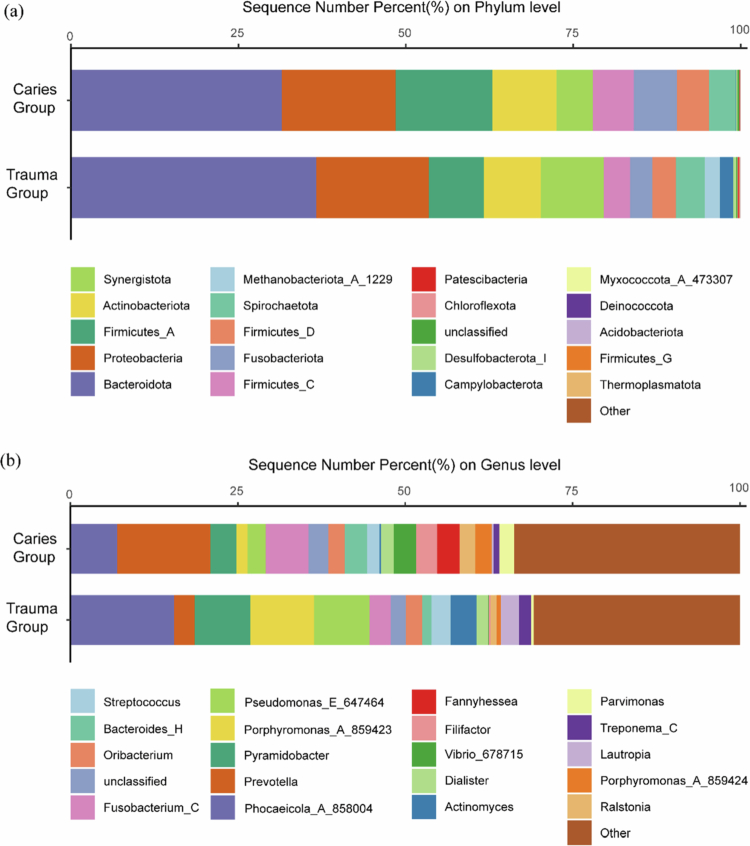
The relative abundance of the predominant bacteria in the inner and outer root apexes of the caries group and the trauma group. (a) Composition of the microbial community at the phylum level; (b) composition of the microbial community at the genus level.

In total, 36 phyla were identified at the phylum level. Among them, the five most abundant phyla and genera were regarded as the dominant ones. The dominant phyla in the caries group were *Bacteroidota* (31.45%), *Proteobacteria* (17.04%), *Firmicutes_A* (14.40%), *Actinobacteriota* (9.58%) and *Fusobacteriota* (6.46%). The dominant phyla in the trauma group were *Bacteroidota* (36.60%), *Proteobacteria* (16.80%), *Synergistota* (9.39%), *Actinobacteriota* (8.55%) and *Firmicutes_A* (8.20%). Figure 2a illustrates the 15 most abundant phyla in the trauma group and the caries group. A total of 587 bacterial genera were identified at the genus level. The most prevalent genera in the caries group were *Prevotella* (13.92%), *Phocaeicola* (6.99%), *Fusobacterium_C* (6.41%), *Pyramidobacter* (3.87%) and *Vibrio* (3.36%). The dominant genera in the trauma group were *Phocaeicola* (15.46%), *Porphyromonas* (9.55%), *Pyramidobacter* (8.31%), *Pseudomonas* (8.26%) and *Actinomyces* (3.86%). Figure 2b illustrates the 15 most prevalent genera in the trauma and caries groups.

### Microbial diversity

**Figure 3.**
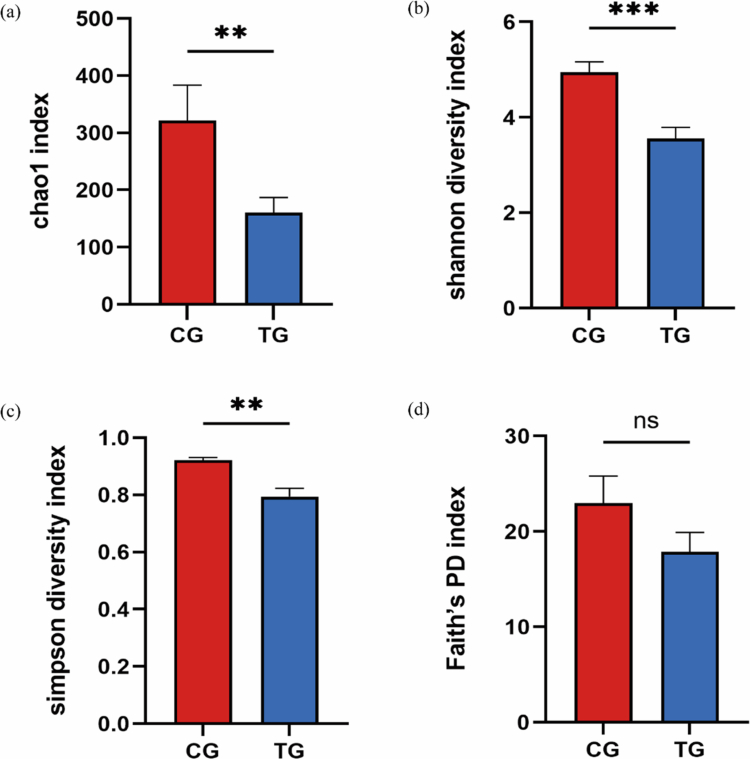
Bacterial alpha diversity analysis in the caries group (CG) and trauma group (TG). (a), (b), (c) and (d) represent the Chao1 index, Shannon index, Simpson index and Faith's phylogenetic diversity (PD) index, respectively. **indicates a large difference compared to the CG group (*P* < 0.01). ***indicates a large difference compared to the CG group (*P *< 0.001). ^ns^indicates no difference compared to the CG group (*P *> 0.05).

Prior to alpha diversity analysis, all samples were subjected to rarefaction, where data with a unified sequencing depth were randomly sampled to standardize the sequencing depth, ensuring the reliability of subsequent diversity index calculations. Figure 3 illustrates the alpha diversity of the trauma group and the caries group. The Shannon, Chao1 and Simpson indices of the trauma group were found to be lower than those of the caries group, suggesting that the alpha diversity of the trauma group was indeed lower (*P* = 0.001). In contrast, the difference in the Faith's PD index between the two groups was not statistically significant (*P* = 0.248), which implies that the phylogenetic diversity of the microorganisms in both groups evolved at a comparable level.

**Figure 4.**
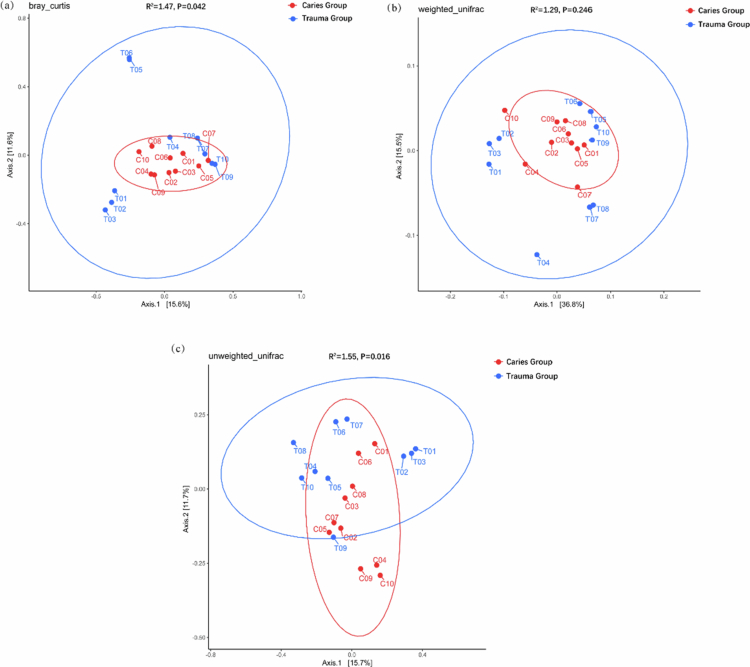
Principal coordinate analysis (PCoA) of the caries group and the trauma group.​ (a), (b) and (c) represent the analysis results based on the Bray‒Curtis distance, weighted UniFrac distance and unweighted UniFrac distance, respectively.

Principal coordinate analysis (PCoA) was performed to assess microbial community structure differences between the trauma group and the caries group, using three distinct distance metrics: Bray‒Curtis, weighted UniFrac and unweighted UniFrac (Figure 4).​ The Bray–Curtis distance, which focuses on species abundance similarity without considering evolutionary relationships, revealed overlapping clustering regions between the two groups. However, permutational multivariate analysis of variance (PERMANOVA) with 999 permutations indicated statistically significant differences in overall microbial community structure (*P* = 0.042).​ The weighted UniFrac distance – an index that integrates species evolutionary relationships and relative abundance, with greater contributions from dominant species – showed no statistically significant difference between the two groups (*P* = 0.246).​ The unweighted UniFrac distance, which is based on species presence/absence and evolutionary relationships and prioritizes rare or endemic species, detected significant statistical differences in low-abundance species composition between the trauma group and caries group (*P* = 0.016).

### Species differences between the trauma group and the caries group

**Figure 5.**
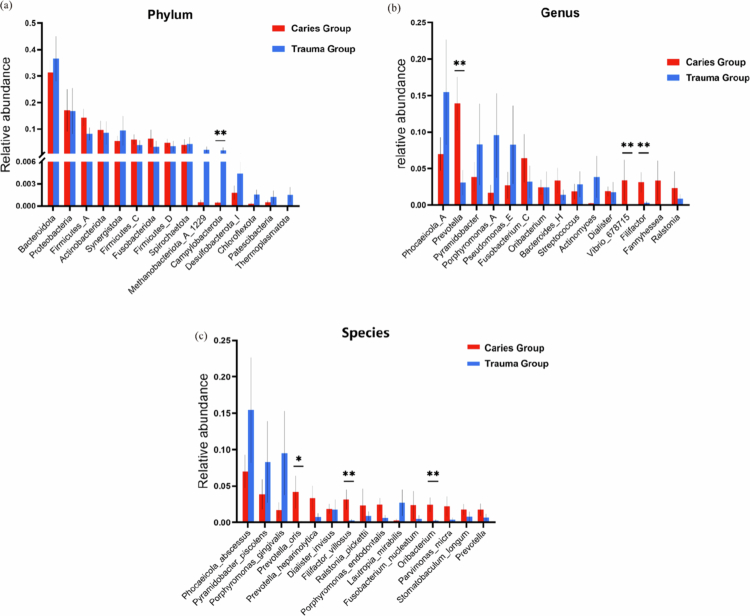
Species differences between caries-derived periapical lesions and trauma-derived periapical lesions were analyzed at the phylum (a), genus (b) and species levels (c). *indicates a highly difference compared to the caries group (*P *< 0.05). **indicates a highly difference compared to the caries group (*P *< 0.01).

The top 15 species with the highest relative abundance were selected separately at the phylum, genus and species levels. Subsequently, a species difference analysis was conducted for the caries group and the trauma group, as shown in Figure 5a–c. The results demonstrated that, at the phylum level, the relative abundance of *Campylobacterota* in the trauma group was greater than that in the caries group, and this difference was statistically significant (*P* = 0.002). Additionally, at the genus level, the relative abundances of *Prevotella*, *Vibrio* and *Filifactor* in the caries group were greater than those in the trauma group, and the differences were statistically significant (*P* = 0.008, 0.041 and 0.006, respectively). At the species level, the relative abundances of *Prevotella oris*, *Filifactor villosus* and *Oribacterium* in the caries group were greater than those in the trauma group, and the differences were statistically significant (*P* = 0.018, 0.007 and 0.004, respectively).

**Figure 6.**
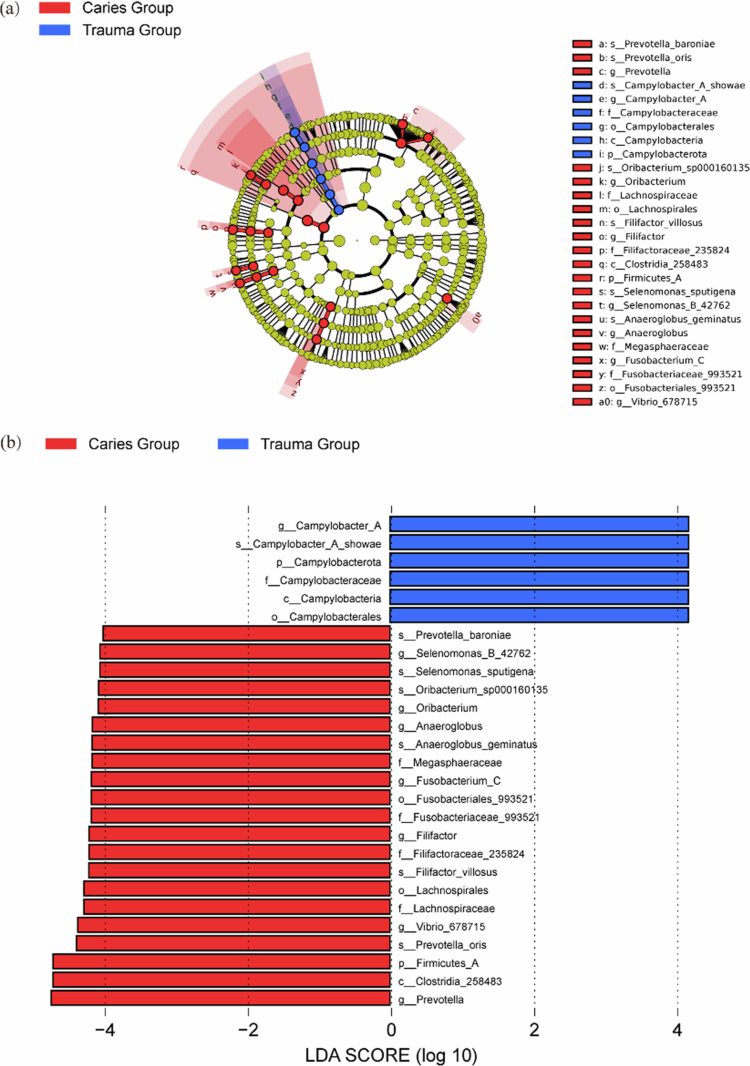
(a) LEfSe evolutionary branch diagram of the caries group and the trauma group. The innermost circle represents the phylum, with each subsequent circle denoting an increasingly specific classification level, namely, class, order, family, genus and species. The lines between the levels indicate the level of affiliation. The circles represent species, with yellow indicating no significant difference between groups. The absence of yellow indicates that the species is a distinctive microorganism within the corresponding colour group, exhibiting a significantly greater abundance within this group. The sectored area denotes the subordinate taxonomic interval of the characteristic microorganism. (b) LDA discriminant histogram. The length of the histogram represents the effect of species abundance on the difference. A longer length indicates a greater effect, and vice versa. The figure illustrates the species that exhibit differential characteristics when the LDA ≥ 2.

As depicted in Figure 6a and 6b, the results of the linear discriminant analysis effect size (LEfSe) difference analysis (LDA ≥ 2) are as follows. At the phylum level, the abundance of *Campylobacterota* in the trauma group was greater than that in the caries group (*P​​​​​​​* *< *0.05). On the contrary, the abundance of *Firmicutes_A* in the caries group was observed to be greater than that in the trauma group (*P​* *< *0.05). At the genus level, the relative abundance of seven genera, namely, *Prevotella*, *Vibrio*, *Filifactor*, *Fusobacterium_C*, *Anaeroglobus*, *Oribacterium* and *Selenomonas*, were greater in the caries group compared to the trauma group. Additionally, the relative abundance of *Campylobacter_A* was greater in the trauma group than in the caries group.

## Discussion

In this study, 16S rRNA sequencing was employed to comparatively characterize the apical microbiomes of trauma-derived and caries-derived periapical lesions. The investigation pursued two objectives: (1) to analyze the structural similarities and ecological differences between these two pathogenic microbial communities and (2) to identify signature microbial compositions and keystone species specifically associated with trauma-derived periapical lesions. Notably, this constitutes the inaugural systematic investigation of the microbiome in trauma-derived periapical lesions, a previously uncharted research domain in oral microbial ecology.

To objectively characterize microbial composition during initial pathogenic progression while minimizing confounding effects from subsequent clinical interventions on pioneer colonization, primary periapical infections were exclusively selected for sampling. Given the technical constraints of non-surgical approaches in simultaneously accessing intra-apical and extra-apical infected tissues, the acquired microbial profiles specifically represent suspended microorganisms within apical confines [7,21]. To overcome the technical constraints inherent in non-surgical sampling approaches, sterile paper points extending beyond the WL were strategically deployed to capture potential extra-foraminal microbiota, thereby reducing mechanical disturbance to periapical structures during specimen collection [22].

In this study, the apical microbial community of trauma-derived periapical lesions was polymicrobial in composition. The predominant bacterial species identified in trauma-derived periapical lesions were *Phocaeicola*, *Porphyromonas*, *Pyramidobacter*, *Pseudomonas* and *Actinomyces*. Reviewing earlier studies, we observed that findings from various investigations into the microbial composition of traumatized teeth are inconsistent. A study on trauma-derived pulp necrosis with or without periapical periodontitis in immature permanent teeth, analyzing intracanal microbial samples before root canal preparation, identified the most prevalent isolates as *Peptostreptococcaceae[Eubacterium]yurii* (8.9%), *Fusobacterium naviforme/nucleatum* (6.4%), *Capnocytophata sp.* (5.8%), *Granulicatella adiacens* (5.1%), *Eikenella corrodens/Kingella denitrificans* (4.5%), *Streptococcus sanguinis* (3.8%), *Slackia exigua* (3.2%) and *Actinomyces sp.* (2.6%) [23]. Another study comparing the microbial composition in root canals between trauma-derived pulp necrosis and non-traumatic pulp necrosis caused by other infections demonstrated that root canals with trauma-derived pulp necrosis also exhibit high microbial diversity, with significant compositional differences between traumatized teeth and non-traumatic teeth with other infectious origins [24]. These discrepancies may be related to sample inclusion criteria, microbial detection methodologies, tooth types and other factors. Furthermore, considerable variability exists in microbial composition across individual cases of traumatized teeth [25,26], as evidenced by the relatively scattered distribution of trauma group samples in our PCoA analysis.

The primary bacterial species detected in trauma-derived periapical lesions maintain high colonization levels in their caries-derived counterparts, differing only in relative abundances. Additionally, substantial spatial overlap in PCoA ordination patterns – with only a marginal statistical difference (based on the Bray‒Curtis distance) and no statistical difference (based on the weighted UniFrac distance) in beta diversity between groups – coupled with constrained statistical power due to limited sample size (*n* = 10/group), supports the convergence of microbial communities between trauma- and caries-derived periapical lesions. This indicates that the bacterial infection of trauma-derived periapical lesions may have a greater origin in the oral environment than solely through anachoresis – bacteria are ‘attracted’ and migrate to the site of damaged or necrotic tissues in the body, where they colonize and proliferate, such as in pulp tissues that have been traumatized or ischemic. The anachoresis hypothesis predominated in 1940s endodontic theory, positing hematogenous microbial colonization of degenerated pulp tissues through circulatory dissemination [27–29]. Subsequently, there is considerable debate among the scientific community regarding the source of microorganisms following dental trauma, particularly when confronting cases involving structurally intact teeth with clinically confirmed pulp necrosis. Grossman et al. inoculated non-oral bacteria into an animal's oral cavity and gingival sulcus while maintaining coronal integrity through controlled traumatic loading, and the bacteria were found in the pulp within weeks post-intervention [30]. Nevertheless, subsequent evidence has systematically displaced this paradigm through compelling microbiological data demonstrating that traumatic pulp infections primarily originate from gingival sulcus/oral cavity biofilms, with hematogenous dissemination playing a negligible role in post-traumatic microbial pathogenesis [30–32].

Furthermore, the total microbial load detected in trauma-derived periapical lesions was significantly lower than in caries-derived counterparts. Alpha diversity analysis revealed reduced microbial richness and diminished evenness in trauma-derived periapical lesions, with species composition was not as complex as that in caries-derived periapical lesions. This ecological divergence may be related to the divergent microbial infiltration pathways between the two groups. The root canal systems in caries-derived periapical lesions is directly connected with the oral environment, while the crown of the tooth in trauma-derived periapical lesions is intact, blocking the direct entry of oral microorganisms into the root canal. After trauma, microcracks of varying severity develop on the surface of the crown [33], and periodontal tissues, such as gingival sulcus and periodontal ligament are also damaged [34,35], which establishes potential infiltration routes for oral microorganisms into the pulpal complex.

Pathological manifestations arise from polymicrobial synergism within the microbiota consortium [36]. Interspecies dynamics determine the overall ecological behavior of the community, and the pathogenic capacity of the community also varies due to different species composition and abundance [37]. According to our experimental results, although there are various shared microorganisms between trauma-derived and caries-derived periapical lesions, the inter-group difference analysis of species composition and LEfSe analysis results show that there are significant differences in some microbial compositions between the two groups. Even for the shared species, their relative abundances also exhibit obvious distinctions between the two groups. Therefore, it is necessary to distinguish between trauma-derived periapical lesions and caries-derived periapical lesions. The ratio of *Firmicutes* to *Bacteroides* is thought to be related to dysbiosis, such as increased abundance of *Firmicutes* or *Bacteroidetes* species associated with obesity and intestinal inflammation, respectively [38]. An elevated ratio of *Firmicutes* to *Bacteroides* may promote oral dysbiosis [39]. The ratio of *Firmicutes* to *Bacteroidetes* in persistent infection root canals is lower than that in primary infected root canals [40]. In this study, the ratio of *Firmicutes* to *Bacteroidetes* in trauma-derived periapical lesions (0.22) was lower than that in caries-derived periapical lesions (0.46), which may suggest that the microbiota ecology of trauma-derived periapical lesions is more stable than that of caries-derived periapical lesions.

In this study, it was observed that *Prevotella*, *Vibrio* and *Filifactor* were significantly lower in trauma-derived periapical lesions compared to caries-derived periapical lesions. The reduction of obligate anaerobic taxa, particularly *Prevotella spp.*, in chronic respiratory pathologies suggests that microbial dysbiosis within the airway microbiome may potentiate pulmonary disease progression through compromised colonization resistance and altered metabolic cross-feeding networks [41]. The relatively isolated microbial community of trauma-derived periapical lesions may create distinct microenvironmental conditions (e.g. pH and oxygen levels) compared to caries-derived periapical lesions, potentially driving a reduction of obligate anaerobic taxa such as *Prevotella*. Nevertheless, it remains to be verified whether these changes in anaerobic bacterial abundance play a role in the pathogenesis of trauma-derived periapical lesions in the context of pulmonary disease progression.

It is noteworthy that *Phocaeicola abscessus*, a Gram-negative, strictly anaerobic bacterium, is highly abundant species in both types of periapical lesions. Previous studies have shown a high detection rate in symptomatic persistent infection root canals [42]. The presence of *Phocaeicola abscessus* in endodontically treated canals with persistent symptoms may be attributed to its resilience to routine endodontic treatment and its capacity to migrate from the primary infected canal.

*Campylobacter* emerged as a discriminative species of trauma-derived periapical lesions through inter-group differential analysis and LEfSe differential analysis. This finding is consistent with a previous study, which also observed a high detection rate of this genus in the root canals of teeth with traumatic pulp necrosis, suggesting a potential specific colonization tendency of *Campylobacter* in trauma-related pulp and periapical infections [24]. *Campylobacter* has been shown to be more abundant in persistently infected root canals than in primary infected root canals [43], with a detection rate of up to 90% [42]. *Campylobacter* exhibits multifunctional virulence mechanisms, including intrinsic antimicrobial resistance [44], biofilm adhesion/formation, cytotoxin secretion and host immune evasion capabilities [45–47], with clinical linkage to aggressive periodontitis progression [48]. *In vitro* studies have revealed that oral *Camplyobacter* exhibits significantly greater biofilm-forming capacity compared to enteric counterparts [49]. A study comparing the efficacy of calcium hydroxide and chlorhexidine gel in regenerative endodontic treatment for traumatic pulp necrosis in immature permanent teeth showed that *Campylobacter* was only detected in root canals after calcium hydroxide dressing. This may suggest that calcium hydroxide has a poor effect on eliminating *Campylobacter* in root canals, though this finding had no impact on the success or failure of regenerative endodontic treatment in that study [23]. Although the *Campylobacter* detected in this study demonstrated subdominant colonization status, certain low-abundance operational taxonomic units (ASVs) exhibited keystone pathogen characteristics capable of inducing dysbiosis. These keystone taxa disproportionately influence community architecture and functional dynamics through non-redundant ecological interactions, independent of their spatiotemporal abundance profiles [50]. Notably, *Porphyromonas gingivalis* functions as a keystone pathogen that elicits microbiome dysbiosis and drives periodontal tissue breakdown even at subthreshold colonization thresholds, demonstrating disproportionate pathogenic influence relative to its abundance through targeted proteolytic activity [50,51]. In light of the findings in this study, targeted investigations are warranted to elucidate whether *Campylobacter* functions as a keystone pathogen in trauma-derived periapical lesions through virulence modulation of the polymicrobial consortium.

It should be noted that this study is not without limitations. 1. In this study, 16S rRNA gene sequencing was restricted to the V3-V4 hypervariable regions. Owing to their limited sequence diversity, these regions hinder the discrimination of some closely related species – particularly taxa with genomic similarity exceeding 97% [52]. In contrast, full-length 16S rRNA gene sequencing can unravel extensive species diversity within the gut microbiome and enable the characterization of several unclassified operational taxonomic units (ASVs) that are absent from amplicon data [53]. This suggests that the V3–V4 regions used in our study may have overlooked certain species-level details. Thus, our analysis focused primarily on differences at the genus and higher taxonomic levels, where the results are more stable under V3–V4 resolution, with species-level findings presented as supplementary references. Future studies integrating full-length 16S sequencing or metagenomics [54,55] could further improve species identification accuracy, especially for distinguishing closely related pathogenic species. Consequently, the findings of this study represent a preliminary investigation into the microbial composition of trauma-derived periapical lesions. 2. The sampling methodology in this study did not encompass microorganisms colonizing anatomically complex niches, including dentinal tubules, the root canal isthmi, and biofilms. 3. Orthodontic cases were classified as trauma in this study, based on mechanical similarities to other types of trauma (e.g. external injury, occlusal trauma) – excessive orthodontic force can damage dental/periodontal tissues (pulp inflammation, PDL inflammation and root resorption), with severity linked to force magnitude, direction and duration [56,57]. However, this classification lacks subtype validation. While we found microbial differences between the combined trauma group (including inappropriate orthodontic treatment) and the caries group, the small sample size limited subgroup analysis (e.g. orthodontic vs. occlusal trauma). In our subsequent studies, we will incorporate etiological details and expand the sample size to conduct subgroup comparisons, thereby improving the accuracy of microbial association analyses.

From the perspective of microbial ecology, although the microbial communities of the trauma group and the caries group are relatively similar at the level of high-abundance species, there are significant differences in the composition of some species (e.g. *Campylobacter*), indicating that the two types of periapical lesions have distinguishable biological bases. These differences in microbial community structure may provide reference indicators for clinical prognosis evaluation and the selection of antimicrobial regimens. It should be noted that this study is only a preliminary observational study. This study provides initial clues for understanding the microbiological differences in chronic apical periodontitis caused by different etiologies, but still needs to be verified by more studies. Therefore, we highly anticipate that future studies will explore the microbial composition of traumatic periapical lesions more comprehensively and deeply, providing stronger support for the formulation of targeted treatment strategies.

## Conclusions

Trauma-derived periapical lesions exhibit lower microbial diversity compared to caries-derived periapical lesions. Notably, significant differences exist in microbial community composition between these etiological types, and *Campylobacter* potentially serves as a characteristic species in trauma-derived periapical lesions.

## Acknowledgments

The authors wish to thank Yunnan Key Laboratory of Stomatology for providing the experimental site and technical support.

## Author contributions

Lijun Huo conceived the design and led the team; Rui She and Shanshan Huang contributed to recruiting patients, conducting clinical treatment and collecting samples; Jia Liang and Fengjiao Zhu are responsible for samples collection, management and transportation; Xinhui Meng, Yiquan Wang completed data collection and preprocessing; Jiyuan Zhan, Yinxue Huang analyzed and visualized the data; Jiyuan Zhan drafted the manuscript; Lijun Huo critically reviewed and revised the manuscript.
